# Whole-exome sequencing in familial type 2 diabetes identifies an atypical missense variant in the RyR2 gene

**DOI:** 10.3389/fendo.2024.1258982

**Published:** 2024-02-20

**Authors:** Vikas Bansal, Bernhard R. Winkelmann, Johannes W. Dietrich, Bernhard O. Boehm

**Affiliations:** ^1^ Department of Pediatrics, University of California San Diego, La Jolla, CA, United States; ^2^ Institute of Genomic Medicine, University of California San Diego, La Jolla, CA, United States; ^3^ ClinPhenomics, Frankfurt am Main, Germany; ^4^ Diabetes, Endocrinology and Metabolism Section, Department of Internal Medicine I, St. Josef Hospital, Ruhr University Hospitals, Bochum, Germany; ^5^ Diabetes Center Bochum-Hattingen, St. Elisabeth-Hospital Blankenstein, Hattingen, Germany; ^6^ Center for Rare Endocrine Diseases, Ruhr Center for Rare Diseases (CeSER), Ruhr University Bochum and Witten/Herdecke University, Bochum, Germany; ^7^ Center for Diabetes Technology, Catholic Hospitals Bochum, Bochum, Germany; ^8^ Lee Kong Chian School of Medicine, Nanyang Technological University, Singapore, Singapore

**Keywords:** type 2 diabetes, RyR2, monogenic diabetes, pathogenic variant, metabolic syndrome, CPVT

## Abstract

Genome-wide association studies have identified several hundred loci associated with type 2 diabetes mellitus (T2DM). Additionally, pathogenic variants in several genes are known to cause monogenic diabetes that overlaps clinically with T2DM. Whole-exome sequencing of related individuals with T2DM is a powerful approach to identify novel high-penetrance disease variants in coding regions of the genome. We performed whole-exome sequencing on four related individuals with T2DM – including one individual diagnosed at the age of 33 years. The individuals were negative for mutations in monogenic diabetes genes, had a strong family history of T2DM, and presented with several characteristics of metabolic syndrome. A missense variant (p.N2291D) in the type 2 ryanodine receptor (*RyR2)* gene was one of eight rare coding variants shared by all individuals. The variant was absent in large population databases and affects a highly conserved amino acid located in a mutational hotspot for pathogenic variants in Catecholaminergic polymorphic ventricular tachycardia (CPVT). Electrocardiogram data did not reveal any cardiac abnormalities except a lower-than-normal resting heart rate (< 60 bpm) in two individuals – a phenotype observed in CPVT individuals with *RyR2* mutations. RyR2-mediated Ca^2+^ release contributes to glucose-mediated insulin secretion and pathogenic *RyR2* mutations cause glucose intolerance in humans and mice. Analysis of glucose tolerance testing data revealed that missense mutations in a CPVT mutation hotspot region – overlapping the p.N2291D variant – are associated with complete penetrance for glucose intolerance. In conclusion, we have identified an atypical missense variant in the *RyR2* gene that co-segregates with diabetes in the absence of overt CPVT.

## Introduction

Diabetes mellitus is a heterogeneous disorder that encompasses several distinct forms each with characteristic clinical presentation at onset and during follow-up (1–3). Type 2 diabetes mellitus is the most common form of diabetes that results from a combination of genetic and environmental risk factors (1, 3). Large-scale genome-wide association studies (GWAS) have identified several hundred distinct T2DM susceptibility loci in the human genome (4). However, disease-associated variants at these loci account for only a small fraction of the heritability of T2DM. The availability of low-cost DNA sequencing technologies has allowed researchers to investigate the contribution of rare variants - particularly in coding regions of the human genome – to risk for T2DM. A number of case-control studies for T2DM that utilize exome sequencing have been published (5, 6). These studies have identified several diabetes-associated rare variants (7, 8) and linked several genes (e.g. SLC30A8) with risk for T2DM (9, p. 30; 5).

In spite of large sample sizes, sequencing based case-control studies have limited ability to link very rare variants with disease due to the low number of carriers of such variants in the dataset. Exome or whole-genome sequencing studies in families with clustering of T2DM have the potential to identify high-penetrance risk variants for diabetes. In this approach, comparison of the DNA sequences of affected and unaffected family members can be used to identify high-penetrance disease variants that are very rare or absent in the population. (10) utilized this strategy to associate loss-of-function variants in the APPL1 gene with diabetes in two families with a high prevalence of diabetes. Similarly, a missense mutation in the beta-cell specific transcription factor MAFA was associated with autosomal dominant diabetes (and insulinomas) using the same approach (11). Such sequencing based studies have a greater chance of success in families with young-onset diabetes or with a multi-generation family history of diabetes. Targeted sequencing studies of monogenic diabetes genes in individuals diagnosed with T2DM have shown that individuals with a lower age at diagnosis have a higher prevalence of pathogenic mutations in MODY genes (12–14). Nevertheless, the vast majority of such individuals are negative for mutations in monogenic diabetes genes suggesting that novel high-penetrance risk variants for T2DM remain to be identified.

In this study, we used whole-exome sequencing to investigate the genetic basis of T2DM in a group of four related individuals identified from a large case-control study of diabetes. Notably, all individuals presented with high BMI and characteristics of metabolic syndrome suggesting the presence of classical T2DM. Additionally, two of the four sequenced individuals were diagnosed with diabetes at the age of 33 and 35 years.

## Materials and methods

### Subjects

The four individuals sequenced in this study were identified from a previous case-control sequencing study for T2D (12). All individuals were residents of Germany and clinical data was obtained from the treating physicians. All individuals were screened for the presence of complications of diabetes (neuropathy, retinopathy, nephropathy). Islet cell-specific autoantibodies (ICA, GADA, IA-2A), were also measured in all individuals to rule out the presence of autoimmune diabetes. Phenotyping included extensive clinical chemistry evaluation (**Supplementary Information**) 12-lead electrocardiogram was performed on all individuals.

### Whole exome sequencing and variant calling

Whole exome sequencing was performed on genomic DNA obtained from the four individuals (Novogene Corporation, Sacramento California) using Agilent SureSelect All Exon V6 exome capture kits and the Illumina sequencing technology. Paired-end sequence reads (150 base pairs in length) for each individual were aligned to the UCSC hg19 reference human genome sequence using the BWA aligner (v 0.7.16). The aligned reads were sorted using samtools and PCR duplicates were marked using Picard (http://broadinstitute.github.io/picard/). Variant calling was performed jointly for the four individuals using the Genome Analysis Toolkit (GATK v4.0.1.2, HaplotypeCaller). Variants showing skewed allele ratio for heterozygous genotypes that overlapped segmental duplications in the human genome were filtered out.

### Variant annotation and association analysis

All identified variants were annotated using the Annovar annotation program and the RefSeq transcript database (15). Computational predictions for missense variants using in-silico prediction tools such as PolyPhen2(16), SIFT (17), MutationAssessor (18), FATHMM (19), Provean (20) and REVEL(21) were obtained from the dbNSFP database (22). Variant allele frequencies were annotated using the Genome Aggregation Database or gnomAD (v2.1.1) (23). Single-variant association statistics were obtained from the Genebass (24) web portal (app.genebass.org). For type 2 diabetes association analysis, we used the phenocode ‘T2D_custom’ with 20813 cases and 371952 controls.

### IBD analysis

Genetic relatedness was estimated using the KING tool (25) that performs identity-by-descent (IBD) analysis for pairs of individuals from unphased genotype data.

### Statistical data analysis

Oral glucose tolerance test (OGTT) data for CPVT patients was obtained from (26). The OGTT values for 27 individuals were divided into two groups based on the location of the mutations and statistical analysis was performed using Python (scipy package).

## Results

The four individuals selected for this study were identified from a targeted sequencing study that was conducted to search for pathogenic variants in genes associated with monogenic and type 2 diabetes (12). All four individuals had diabetes that was clinically classified as T2DM. Sharing of rare variants identified from the targeted sequencing data indicated that the individuals were related to each other. One of the four individuals – individual II - was diagnosed with diabetes at the age of 33 years (**Table 1**) and had a BMI of 24.8 at diagnosis. Individual III was diagnosed with diabetes at the age of 35 years and was obese (BMI = 32.1) at the time of diagnosis with increasing body weight since age 21. The two other individuals – I and IV - were diagnosed with diabetes at the ages of 45 and 47 respectively. All individuals reported a positive family history of diabetes (one or more siblings afflicted with diabetes). Hypertension was present in all four individuals and 3 of the 4 individuals also had low HDL cholesterol levels (**Table 1**). Therefore, the individuals presented with several clinical features of metabolic syndrome. None of the individuals were positive for islet cell antibodies, namely ICA, GADA and IA-2A ruling out a role of islet-cell autoimmunity as a driver of diabetes manifestation. Analysis of insulin-glucose homeostasis showed that the secretory capacity of beta-cells at the fasting state was higher in two of the four individuals (individuals II and IV) compared to normoglycemic subjects, suggesting a compensatory increase of insulin release (**Supplementary Tables 1, 2**). Individual III had evidence of glucose intolerance prior to diagnosis of diabetes (2 hour oral glucose tolerance test (OGTT) value equal to 193 mg/dl). At diagnosis, this individual had elevated C-peptide (3.8 ug/l) and proinsulin levels (30 pmol/l) – indicative of an insulin resistant state. The two hour OGTT also revealed an abnormal insulin response with high proinsulin to insulin ratio at 120 minutes (**Supplementary Figure 1**). In particular, the elevated two-hour proinsulin indicated a high level of ER stress at the level of the beta cells.

**Table 1:** Phenotypes for the four individuals sequenced in the study.

	I	II	III	IV	*Normal range*
**Gender**	Male	Male	Female	Male	
**Age at DM diagnosis** **(years)**	45	33	35	47	
**BMI at DM diagnosis** **(kg/m²)**	27.1	28.7	32.1	32.5	18.5 – 24.9
**Hypertension**	Yes	Yes	Yes	Yes	
**HDL-C** **(mg/dl)**	34	35	43	39	40-60
**TG** **(mg/dl)**	103	431	101	214	< 150
**HbA1c (%)**	6.3	7.2	5.4	7.5	< 5.7

DM, diabetes mellitus; BMI, body mass index in kg/m^2^; TG, triglycerides. The phenotypes were obtained at the time of recruitment.

The individuals were previously observed to be negative for any potential pathogenic variants in commonly mutated MODY genes(12). Therefore, we chose to perform exome sequencing to search for rare coding variants that potentially contribute to the shared diabetic phenotype. Whole-exome sequencing on the four individuals using paired-end Illumina sequencing technology (Methods) resulted in 6-7 gigabases of aligned sequence data and 58-65x coverage of the targeted regions (**Supplementary Table 3**). Variant calling using the reads aligned to the reference human genome (hg19) – jointly for the four individuals - identified approximately 360,000 single nucleotide variants and short insertion/deletion variants (see Methods).

**Figure 1:**
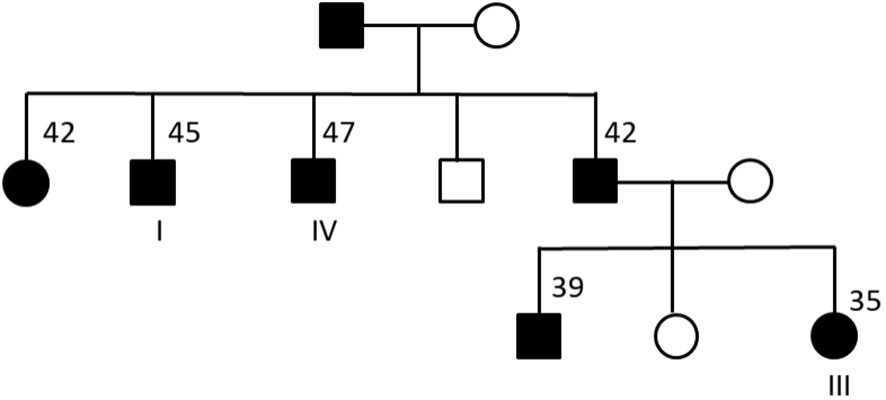
Family tree for three of the four sequenced individuals. Males are represented using squares and females by circles. Black filled symbols correspond to individuals with type 2 diabetes. For some of the individuals, the age at diagnosis of diabetes is shown. Individual II (not shown) was inferred to be a sibling of individuals I and IV based on IBD analysis.

First, we performed identical-by-descent (IBD) analysis using the variants identified from the exome sequence data. IBD analysis using the KING tool for analyzing genetic relatedness (25) confirmed that the four individuals are related: individuals I, II and IV had pairwise IBD estimates (IBD1 = 0.43-0.45 and IBD2 = 0.24-0.26) that are typically seen in full siblings (**Supplementary Table 4**). Individual III also had high IBD sharing (IBD1 = 0.33 – 0.46) with the other three individuals indicative of a second-degree relationship. IBD analysis using another tool for estimating genetic relatedness (27) produced very similar results (data not shown). Next, we contacted the physicians for these four individuals and obtained family trees for the individuals. The family trees confirmed that individual III shared an avuncular relationship with individuals I and IV (**Figure 1**). Notably, individual III’s father (sibling of individuals I and IV) and paternal grandfather also has diabetes consistent with a dominant mode of inheritance for diabetes. Although we could not obtain full family data for individual II, IBD based inference clearly indicated that individual II is a sibling of individuals I and IV.

To search for rare genetic variant(s) that could explain the presence of diabetes in the four individuals under a dominant model of inheritance, we used a standard filtering approach for prioritizing variants identified from the exome sequencing (28). First, we filtered out variants present at a minor allele frequency of 0.1% or greater in the gnomAD database (variant data from 125,748 exomes and 15,708 genomes). Next, we prioritized variants that were predicted to affect the protein coding sequence (missense, frameshift or stop-loss) or impact splicing. There were only eight such variants (**Table 2**) – all missense variants - that were shared by all four sequenced individuals. Two of these missense variants (p.N2291D in *RyR2* and p.S33R in *SLC30A9*) were absent in the gnomAD database. Based on the family tree (**Figure 1**), we can infer that each of the eight missense variants is also present in the diabetic father of individual III and absent in the unaffected mother of individual III.

**Table 2:** List of rare protein-impacting variants shared by all sequenced individuals.

Variant coordinates^a^	Gene	Variant annotation	Coding impact	gnomAD allele frequency^b^
1:237801735:A:G	*RYR2*	missense	NM_001035:c.A6871G:p.N2291D	–
16:20788827:G:C	*ACSM3*	missense	NM_005622:c.G563C:p.C188S	0.00005 (13/128766)
17:40734132:G:C	*RETREG3 (FAM134C)*	missense	NM_178126:c.C1100G:p.P367R	0.00017 (43/128882)
20:34761811:G:A	*EPB41L1*	missense	NM_012156:c.G112A:p.G38S	0.00006 (3/128114)
3:73439024:G:C	*PDZRN3*	missense	NM_001303139:c.C453G:p.D151E	0.00068 (88/129182)
7:122261764:T:C	*CADPS2*	missense	NM_001009571:c.A875G:p.K292R	0.00063 (131/119776)
17:39616396:A:G	*KRT32*	missense	NM_002278:c.T1313C:p.M438T	0.001 (176/127730)
4:41992765:A:C	*SLC30A9*	missense	NM_006345:c.A97C:p.S33R	–

a The variant coordinates (chromosome:position:reference_allele:alternate_allele) are provided with respect to the hg19 human reference genome sequence.

b The overall allele frequency from gnomAD is reported along with the allele counts (variant/total) in European (non-Finnish) population in brackets.

Three of the eight genes with missense variants – *RYR2*, *ASMC3* and *CADPS2* – had evidence of prior association with diabetes-relevant phenotypes or metabolic syndrome in humans or model organisms. *RYR2* is expressed in the pancreatic beta cells and known to mediate Ca^2+^ release that plays an important role in glucose-mediated insulin secretion (29, 30). Similarly, *CADPS2* is also expressed in the pancreatic beta cells and CADPS2^-/-^ mice show reduced glucose-induced insulin secretion (31). *ACSM3* (acyl-CoA synthetase medium chain family member 3) is highly expressed in the liver and kidneys and was identified as a candidate gene that may contribute to hypertension in rats and humans (32, 33). Another gene with a missense variant, *SLC30A9*, is known to cause a rare recessive disease with complex movement abnormalities and renal impairment (34, 35). *SLC30A9* is a member of the SLC30 family of zinc transporters and variants in a different member of this family (*SLC30A8*) have been associated with risk of T2DM (9).

Next, we used predictions from several individual in-silico missense prediction tools (PolyPhen2, SIFT, MutationAssessor, Provean and FATHMM) and the ensemble predictor REVEL (21) to analyze the deleteriousness of the eight missense variants. REVEL combines predictions from 13 individual tools into a single score (ranging from 0 to 1 with higher scores corresponding to greater chance of pathogenicity) and has been shown to be highly effective in discriminating pathogenic variants from benign variants. The missense variant in *RYR2* had the highest REVEL score equal to 0.783 followed by the missense variant in *ACMS3* (0.304, **Supplementary Table 5**). The missense variants in *RYR2* and *ACMS3* were the only variants that were predicted to be deleterious by at least three of the five individual prediction tools (**Supplementary Table 5**). In contrast, the missense variants in the *CADPS2* and *SLC30A9* genes were predicted to be benign by at least four of the five tools and had low REVEL scores (< 0.1).

**Figure 2:**
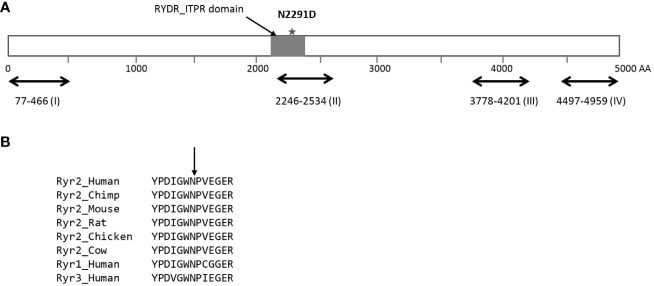
Location of the p.N2291D missense variant in the sequence of the RyR2 protein. **(A)** Schematic of the Ryr2 protein (4967 residues long) with the four hotposts for missense mutations observed in individuals with CPVT. The p.N2291D is located in the RIH (RyR and IP3R Homology) domain of the protein and also overlaps hotspot II. **(B)** The asparagine residue at position 2291 is conserved across the RyR2 protein sequence of multiple species and also across the RyR gene family in humans.

*RYR2* is a well-known Mendelian disease gene that encodes for the cardiac ryanodine receptor - the major calcium release channel on the sarcoplasmic reticulum in cardiomyocytes (36, 37, p. 2). Heterozygous mutations in *RYR2* cause cardiac arrhythmias including Catecholaminergic polymorphic ventricular tachycardia (CPVT) that are characterized by exercise-induced syncope, sudden cardiac arrest or sudden cardiac death. Pathogenic CPVT mutations in *RYR2* cluster in four hotspots and the p.N2291D variant is located in the second mutational hotspot (AA 2246-2534, **Figure 2A**). The N2291 residue overlaps the RIH (RyR and IP3R Homology) domain of the protein and is conserved across species and across the paralogs of the ryanodine receptor gene family (**Figure 2B**). The p.N2291D variant has been not reported in individuals with CPVT or RyR2-related conditions, however, Clinvar contained a single report (rs1681101676) of the variant being detected in clinical testing for CPVT. The variant was classified as a Variant of Uncertain Significance (VUS) due to insufficient evidence.

There was no reported history of any CPVT-relevant phenotypes such as syncope, sudden cardiac arrest or premature cardiac death triggered by exercise or stress in the four sequenced individuals or their family members. To search for the presence of any subtle cardiac abnormalities, we obtained resting 12-lead electrocardiogram (ECG) data for each of the four individuals. The ECG was normal for individuals I, III and IV (**Table 3**). For individual II, the ECG showed the presence of coronary artery disease (first diagnosed at age 52 with a stroke at age 55) but no other abnormalities. Individuals with CPVT typically have a normal resting ECG but male patients have a significantly lower heart rate compared to unaffected family members (37, 38). In addition, low-frequency variants at the *RYR2* gene locus have been strongly associated with resting heart rate in a genome-wide association study with more than 450,000 individuals (39). The resting heart rate for individuals II and IV – both males - was 55 and 56 bpm respectively, lower than the normal range of 60-100 bpm. The resting heart rates for individual I (male) and III (female) were 60 and 75 bpm respectively. None of the individuals with a low heart rate (individuals I, II and IV) were using beta-blocker medications. Therefore, the lower-than-normal resting heart rate suggested the presence of a mild cardiac phenotype.

**Table 3:** Cardiac phenotypes for the four sequenced individuals obtained from a 12-lead electrocardiogram.

	I (male)	II (male)	III (female)	IV (male)	Normal range
Heart rate	60 bpm	55 bpm	75 bpm	56 bpm	60 - 100 bpm
QRS interval	102 ms	108 ms	90 ms	98 ms	80-120 ms
Sinus rhythm	Yes	Yes	Yes	Yes	
QTc interval	392 ms	400 ms	350 ms	400 ms	see below^1^
PQ interval	243 ms	184 ms	142 ms	180 ms	
P wave	118 ms	143 ms	120 ms	94 ms	
RR	996 ms	1040 ms	786 ms	1054	
ST segment abnormalities	None	None	None	None	No abnormalities
Pathological Q wave	None	Yes	None	None	No abnormalities
Sokolow-Lyon-Index	1.55 mV	2.20 mV	2.20 mV	2.17 mV	< 3.5 mV
Summary	Normal	Q wave and negative T in III – presence of coronary heart disease	Normal	Normal	

Bpm, beats per minute; ms, milliseconds.

^1^An “abnormal” QTc in males is a QTc above 450 ms; and, in females, above 470 ms.

**Figure 3:**
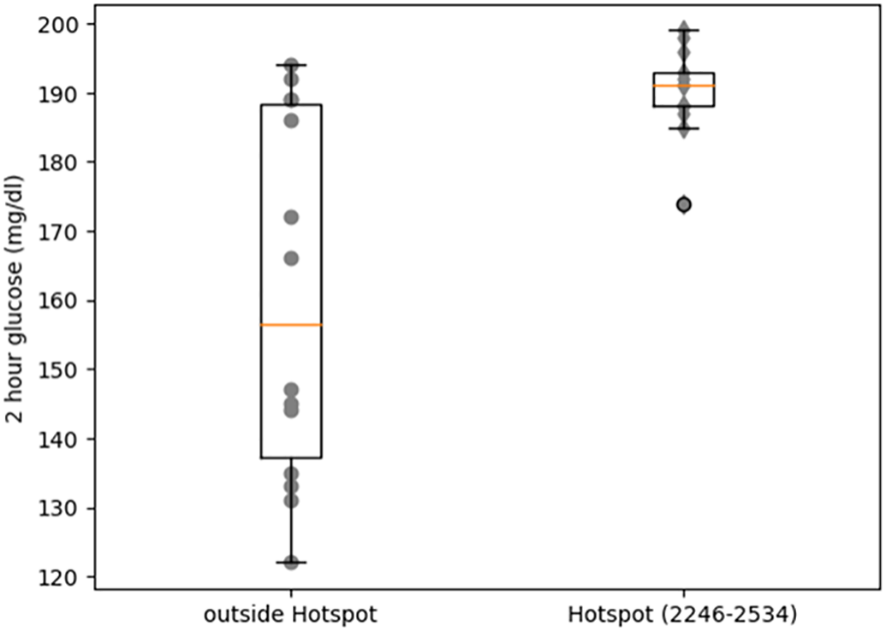
Comparison of blood glucose levels (2h hours after OGTT) between CPVT individuals with mutations in a CPVT hotspot region of *RyR2* (residues 2246 to 2534) and individuals with mutations outside this region.

Heterozygous missense mutations in *RYR2* that cause CPVT result in a leaky RyR2 channel that affects glucose homeostasis in both humans and mice (26). We re-analyzed OGTT data for 27 CPVT patients with heterozygous missense variants in *RYR2* (26) to further dissect the impact of missense mutations in *RYR2* on glucose homeostasis. Stratifying the data based on position of the missense mutations revealed that individuals with *RYR2* mutations in the hotspot II region (**Figure 3**) had significantly higher 2-hour OGTT values (190 ± 6.20) compared to individuals with *RYR2* mutations in the rest of the protein sequence (160.36 ± 25.36, Wilcoxon rank sum test p-value = 0.0048). Notably, 13 of 13 carriers with missense variants in hotspot II region had 2-hour OGTT values of 170 mg/dl or greater and the p.N2291D missense variant was located very close to a cluster of such variants (**Supplementary Figure 2**). This indicated that the second hotspot region of *RYR2* has particular functional importance in pancreatic beta cells and provided additional evidence that the p.N2291D variant is deleterious. In addition, 4 of the 8 individuals with mutations in the C-terminal region (**Supplementary Figure 2**) had normal glucose tolerance (< 140 mg/dl) indicating incomplete penetrance for glucose intolerance due to CPVT-causing mutations.

Finally, we explored the possibility that the *ACSM3* missense variant is a genetic risk factor for diabetes and related phenotypes (e.g. hypertension) in the four individuals. For this, we utilized the Genebass resource of exome-based association statistics for 394,841 individuals from the UK Biobank with 4,529 phenotypes (see Methods). In this dataset, the minor allele frequency of the *ACSM3* variant was 0.00015 similar to the minor allele frequency in the European ancestry individuals in the gnomAD database (0.0001). The variant showed modest association with type 2 diabetes (p-value = 0.0023) but no association with hypertension, HDL cholesterol or BMI (p-value > 0.05). There were 4517 single variant associations available for this variant and the association with type 2 diabetes was not reported to be statistically significant in Genebass. Nevertheless, we cannot rule out the possibility that the *ACSM3* variant is a genetic risk factor for diabetes in the individuals in our study.

## Discussion

In this study, we used whole-exome sequencing to search for rare coding variants involved in susceptibility to diabetes in four related individuals that were clinically classified as T2DM. Several clinical features of metabolic syndrome including hypertension, high BMI, low HDL cholesterol and insulin resistance were also present in the four individuals. We identified a missense variant in the *RyR2* gene that co-segregated with diabetes in six individuals – four sequenced individuals and two non-genotyped individuals (parents of one of the four sequenced individuals). Several lines of evidence indicate that this *RyR2* missense variant is functionally deleterious and likely involved in the pathogenesis of T2DM. First, the variant affects a highly conserved amino acid and is predicted to be deleterious by multiple computational prediction tools. Second, the variant is located in a hotspot for CPVT mutations in *RYR2* that is associated with complete penetrance for glucose intolerance. Finally, the known association of common and rare *RYR2* variants with resting heart rate and the presence of a lower-than-normal heart rate in two of the four individuals suggests that the missense variant is likely functional.

Ryanodine receptors are a family of intracellular Ca^2+^ permeable channels that provide the sarcoplasmic reticulum Ca^2+^ release in various tissues such as skeletal and cardiac muscle. Humans have three such receptors (RyR1-3) and the mutations in all three receptors have been shown to cause Mendelian diseases – both dominant and recessive (30, 40). The type 2 ryanodine receptor is expressed mostly in the heart, brain and pancreas and is expressed at much higher levels than *RyR1* and *RyR3* in islet cells (29). Consistent with these expression patterns, *RyR2* mutations have been linked to phenotypes in each of these tissues. The association of gain-of-function and loss-of-function variants in *RyR2* with cardiac arrhythmias and sudden death is well established (41, p. 2). A recent study linked a *RyR2* missense variant with epilepsy in a 32-year old female without cardiac abnormalities while her brother – carrying the same mutation – had CPVT (42). Another study found an association between missense variants in Ryr2 and epilepsy with or without arrhythmia (43). Dixit et al. used a mouse model to demonstrate that a gain-of-function missense mutation in *RyR2* that results in constitutive phosphorylation led to basal hyperinsulinemia and other known hallmarks of pre-diabetes (29). Santulli et al. demonstrated that CPVT mutations in *RyR2* result in glucose intolerance and impaired insulin secretion in humans and mice (26).

The aforementioned study is the only study that has investigated the association of *RyR2* missense variants with human phenotypes relevant to diabetes (26). 85% of individuals with CPVT and RyR2 mutations in this study were found to have 2-hour impaired glucose levels. Notably, none of the individuals in this study had abnormal fasting glucose or met the criteria for being diabetic (glucose level > 200 mg/dl at 2-hours post OGTT). Our analysis of this data revealed positional heterogeneity in the penetrance of *RyR2* mutations where missense mutations located in one of the hotspots for CPVT mutations (residues 2246 to 2534) had complete penetrance for glucose intolerance while mutations in the C-terminal region had incomplete penetrance. RyR2 is a large protein (4967 residues long) and several hundred pathogenic missense mutations in the gene have been reported. A recent study found that pathogenic variants in *RyR2* associated with sudden death during sleep were enriched in the C-terminal region of the protein (41). Another study that analyzed the prevalence of cardiac phenotypes in relatives of individuals with CPVT found that mutation location was associated with severity of cardiac phenotypes (44). Our results show that this variation in phenotype severity as a function of mutation location for the *RYR2* gene is not limited to cardiac phenotypes.

Type 2 diabetes is known to result from gene-environment interplay. Unlike mutations in monogenic diabetes genes, pathogenic missense variants in *RyR2* result in normal fasting glucose levels with impaired glucose tolerance at an early age (26). However, it is well known that not all individuals with impaired glucose tolerance progress to diabetes at a later age (45). This may explain why diabetes has not been reported to aggregate in families with CPVT. The four individuals with the *RyR2* missense variant presented with a high BMI (28-33 kg/m^2^) and hypertension from an early age. One of the four individuals had impaired glucose tolerance before diagnosis of diabetes and clear evidence of ER stress at the level of the beta cells. Therefore, it is likely that the genetic predisposition in the form of glucose intolerance combined with environmental factors played an important role in progression to clinically overt diabetes.

Our study shows the great potential of combining genetic analysis and phenotyping of subjects clinically classified as type 2 diabetics to pinpoint the heterogeneity of chronic hyperglycemia as an example of precision diabetes medicine in advanced clinical practice (46, 47). Individuals in our study were not recruited as a family but identified from genetic analysis of relatedness among individuals in a case-control study of T2DM. None of the individuals presented at diagnosis as a normal weight individual. All of them had additional phenotypes observed in metabolic syndrome and such individuals are less likely to be included in studies of monogenic diabetes.

This study has a number of limitations. First, we did not evaluate the impact of the missense variant identified in this study on RyR2 channel function. Second, the individuals sequenced in our study were ascertained from a case-control study of diabetes and additional family members could not be included for genotype-phenotype analysis. Finally, we did not evaluate the contribution of common variants associated with risk of T2D or non-coding variants that are not identified in exome sequencing. Further studies are needed to investigate the contribution of rare *RyR2* missense mutations to diabetes and related phenotypes.

## Data availability statement

The data analyzed in the study have been deposited in the Figshare repository, accession number 10.6084/m9.figshare.25044389.

## Ethics statement

The studies involving humans were approved by Institutional Review Board (IRB) of Ulm University, Ulm, Germany; (registration numbers 42/2004 and 189/2007) and the Chamber of Physicians, State Baden-Wuerttemberg, Germany (registration number 133-2002). The studies were conducted in accordance with the local legislation and institutional requirements. The human DNA samples used in this study were acquired from our previous studies for which ethical approval was obtained. All individuals provided written informed consent for using their DNA samples for genetic studies.

## Author contributions

VB: Conceptualization, Investigation, Methodology, Writing – original draft. BW: Data curation, Resources, Writing – review & editing. JD: Data curation, Resources, Writing – review & editing. BB: Data curation, Investigation, Resources, Supervision, Writing – review & editing.
