# Supplementary material for: Whole-exome sequencing in familial type 2 diabetes identifies an atypical missense variant in the RyR2 gene
Source: Front Endocrinol (Lausanne). 2024 Feb 20;15:1258982. doi: 10.3389/fendo.2024.1258982 (PMC10913019; doi:10.3389/fendo.2024.1258982)
Supplement: Supplementary file 1 [file DataSheet_1.pdf]

Supplementary Figures and Tables

Supp Table 1: Analysis of insulin-glucose homeostasis in carriers of RYR2 variants. Standard clinical chemistry parameters were determined using Cobas assay systems (Roche Diagnostics, Penzberg, Germany). Glycated hemoglobin was measured with an immunoassay (Roche Diagnostics, Penzberg, Germany). Intact proinsulin was determined using a proinsulin ELISA (DRG Instruments, Marburg, Germany). Results from the NHANES 2007/2008 cohort were used as an additional reference for the comparative analysis. HOMA-beta, HOMA-IR, SPINA-GR, and QUICKI values were calculated as described previously (see references).

Individual	Fasting glucose concentration (mmol/L)	Fasting insulin concentration (pmol/L)	SPINA-GBeta (pmol/s)	HOMA-Beta (%)	SPINA-GR (mol/s)	HOMA-IR	HOMA-IS	QUICKI
II	15.1	318	7.91	91.42	0.09	35.56	0.03	0.24
I	3.59	78	3.91	2752.94	2.37	2.08	0.48	0.34
III**	5.85	78	2.91	110.64	1.30	3.38	0.30	0.32
IV	10.62	210	5.92	98.28	0.22	16.52	0.06	0.26
Reference range	< 5.6	≤ 137.6	0.65–3,69*	N/A	1,45–8,38*	< 2.5	> 0.4	> 0.4

* NHANES 2009/2010; ** newly diagnosed diabetes

Supp Table 2: **Comparison of beta cell function and insulin resistance in the four individuals with exome sequencing (n=4) and normoglycemic subjects (n=234).** A cohort of 234 normoglycemic subjects (age range: 25-50 years) from the southern part of Germany was used to define 95% confidence intervals of insulin, intact proinsulin and C-peptide values.

Parameter	RYR2 gene variant (n = 4)	Controls (n = 234)	p (t test)	p (Wilcoxon-Mann-Whitney test)
HOMA-Beta	763.3 ± 663.2	91.3 ± 4.6	0.2256 (log†)	0.0684
SPINA-GBeta	5.16 ± 1.11	2.96 ± 0.14	**0.0374** (log†)**	**0.0372****
SPINA-GR	1.00 ± 0.53	1.77 ± 0.11	0.3667 (log†)	0.2872
HOMA-IR	14.38 ± 7.77	5,39 ± 0.31	0.3089 (log†)	0.2205
HOMA-IS	0.22 ± 0.11	0.43 ± 0.03	0.3089 (log†)	0.2205
QUICKI	0.29 ± 0.02	0.32 ± 0.00	0.2778	0.2205

*mean ± SEM; **p < 0.05; †calculated from log-transformed data due to positive skewness

Supp Table 3. Sequencing and coverage metrics for the four individuals sequenced using whole-exome sequencing. Agilent Sureselect V6 target regions were used for analyzing depth using the mosdepth tool (https://github.com/brentp/mosdepth).

**Individual**	**Sequenced bases (Gb)**	**mapped reads (%)**	**Mean depth (targeted regions)**	**>= 10x coverage[a]**
I 0566	6.6	99.90	62.18	95.76
II 0968	6.3	99.80	58.54	95.04
III 0976	6.7	99.91	60.81	95.75
IV 0983	6.9	99.88	64.08	95.72

[a]percentage of targeted bases covered by at least 10 reads

Supp Table 4. Pairwise IBD sharing for the four individuals estimated using whole-exome sequence data and the KING relationship inference tool.

**ID1**	**ID2**	**p.IBD1 estimate**	**p.IBD2 estimate**	**IBD estimate**	**Inferred relationship**
I	II	0.462	0.216	0.447	Full-siblings
I	IV	0.519	0.244	0.504	Full-siblings
II	IV	0.405	0.267	0.470	Full-siblings
I	III	0.427	0	0.213	2nd degree
II	III	0.582	0	0.291	2nd degree
III	IV	0.541	0	0.271	2nd degree

Supp Table 5. In-silico predictions of deleteriousness for the eight missense variants using five different individual computational tools and the ensemble predictor REVEL.

Variant	PolyPhen2[2] (HumDiv/HumVar)	SIFT	Mutation Taster	Provean	FATHMM	REVEL score
RyR2 (N2291D)[1]	D/D	T	D	D	D	0.783
ACSM3 (C188S)	D/P	T	D	D	T	0.304
RETREG3 (P367R)	B/B	T	N	N	T	0.065
EPB41L1 (G38S)	B/B	T	D	N	D	0.140
PDZRN3 (D151E)	D/D	T	D	N	T	0.183
CADPS2 (K292R)	B/B	T	D	N	T	0.066
KRT32 (M438T)	B/B	T	N	N	D	0.130
SLC30A9 (S33R)	B/B	T	N	N	T	0.033

[a]the SIFT alignments included the Drosophila and C. elegans paralog RYR gene sequence which has the ‘D’ amino acid at position 2291 and therefore the N2291D substitution is predicted to be tolerated

Supp Figure 1. Results of the oral glucose tolerance test in individual III performed after diagnosis of diabetes and comparison with non-diabetic individuals. A 75-g OGTT was performed according to WHO recommendations after a 12-hour overnight fast and measurements taken at 0, 30, 60, 90 and 120 minutes.

	0 min	30 min	60 min	90 min	120 min
Glucose (mg/dl)	105	153	212	197	200
Insulin (mIU/l)	13	66	128	109	134
Proinsulin (pmol/L)	30*	139	213	210	315
C-peptide (ug/l)	3.8	8.8	12.2	12.6	12.2

The figure below shows the Insulin (panel A) and proinsulin (panel B) concentrations after oral glucose tolerance testing in individual III compared with non-diabetic subjects**.** Dots correspond to insulin and proinsulin plasma levels during oral glucose tolerance test. Shaded areas give 95% confidence interval of insulin and proinsulin levels of 234 non-diabetic control subjects.

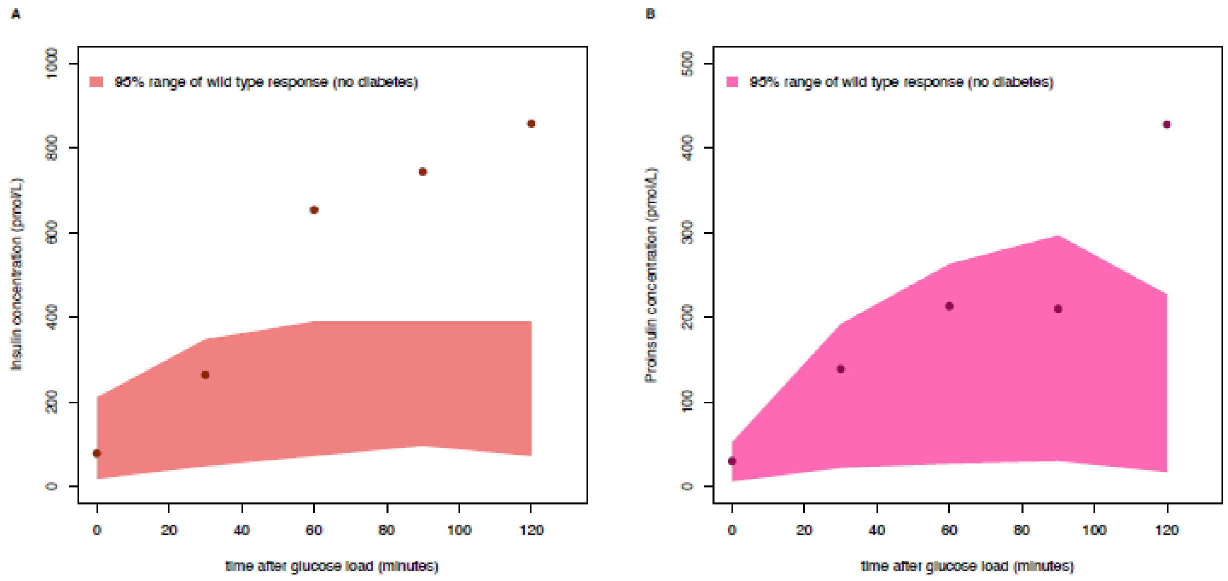

Supp Figure 2. Visualization of 2 hour glucose levels in 27 CPVT individuals with *RYR2* missense mutations as a function of the position of the mutation. The p.N2291D missense variant identified in the current study is shown in red (diamond)

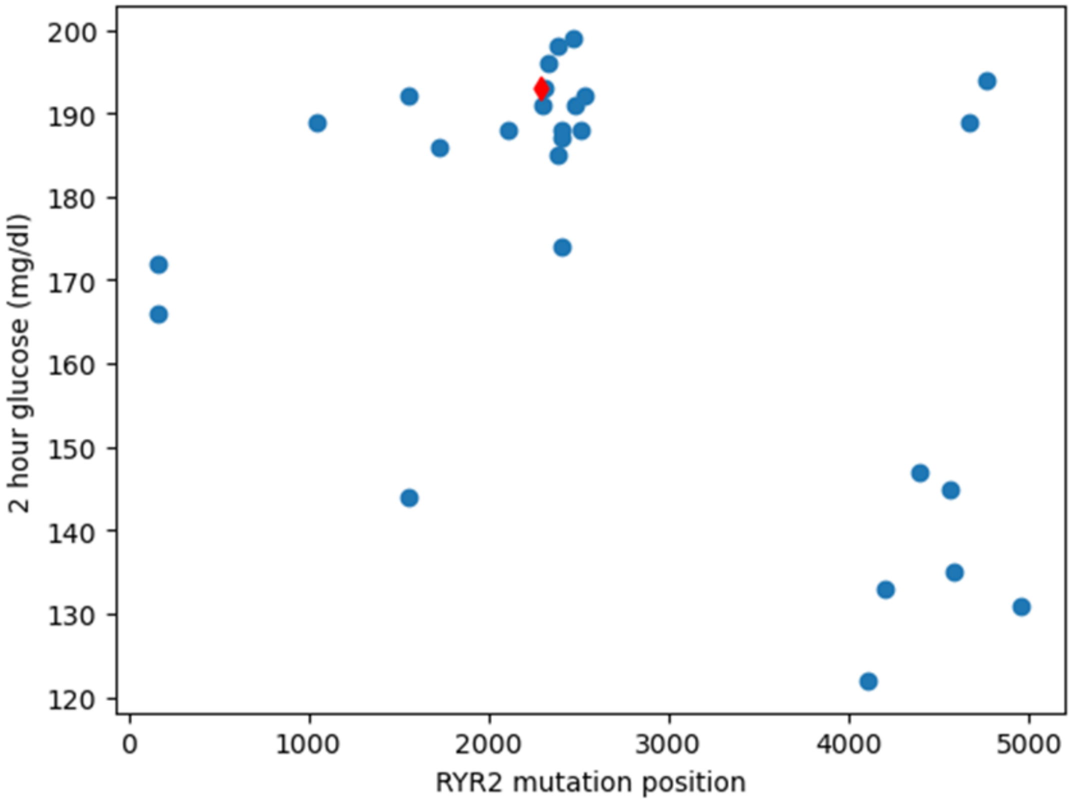

References

1. Dietrich JW, Dasgupta R, Anoop S, Jebasingh F, Kurian ME, Inbakumari M, et al. SPINA Carb: a simple mathematical model supporting fast in-vivo estimation of insulin sensitivity and beta cell function. Sci Rep (2022) 12(1):17659. Epub 2022/10/23. doi: 10.1038/s41598-022-22531-3.

2. Matthews, D. R. et al. Homeostasis model assessment: Insulin resistance and beta-cell function from fasting plasma glucose and insulin concentrations in man. Diabetologia 28, 412–419 (1985).

3. Quantitative insulin sensitivity check index (QUICKI): Katz, A. et al. Quantitative insulin sensitivity check index: A simple, accurate method for assessing insulin sensitivity in humans. J. Clin. Endocrinol. Metab. 85, 2402–2410. https://doi.org/10.1210/jcem.85.7.6661 (2000)

4.Pfützner A, Forst T. Elevated intact proinsulin levels are indicative of Beta-cell dysfunction, insulin resistance, and cardiovascular risk: impact of the antidiabetic agent pioglitazone. J Diabetes Sci Technol. 2011;5(3):784-793. doi:10.1177/193229681100500333

5. Russo GT, Giorda CB, Cercone S, Nicolucci A, Cucinotta D, on behalf of BetaDecline Study Group (2014) Factors Associated with Beta-Cell Dysfunction in Type 2 Diabetes: The BETADECLINE Study. PLoS ONE 9(10): e109702. doi.org/10.1371/journal.pone.010970
